# END-OF-LIFE HOSPITALIZATION SPENDING IN THE LAST SIX MONTHS OF LIFE AMONG CANCER PATIENTS

**DOI:** 10.1093/geroni/igac059.3062

**Published:** 2022-12-20

**Authors:** Katika Akksilp, Vanessa Tan, Julian Lim, Keith Lim, Cynthia Chen

**Affiliations:** National University of Singapore, Singapore, Singapore; National University of Singapore, Singapore, Singapore; Saw Swee Hock School of Public Health, Singapore, Singapore; National University Hospital, Singapore, Singapore; National University of Singapore, Singapore, Singapore

## Abstract

End-of-life care may constitute a vast proportion of health care expenditure globally. In the US, one-quarter of all medicare spending goes towards healthcare for people near the end of life. This is especially true for cancer patients. Our study aimed to estimate hospital spending in cancer patients in the last six months of life. Patients with cancer who died between June 2015 to December 2016 (n=653) were extracted from Singapore’s electronic medical records. A two-part model was used to estimate hospitalization spending. In the last month of life, we found that 85% of the patients did not have chemotherapy, 43% had good quality care, and 31% had palliative care. The average total hospitalization spending in the last six months of life was S$59,180. Further analysis by month from death revealed that spending increased sharply by 62%, from $7,591 in the sixth month of life to $12,315 in the last month of life. Decedents who received chemotherapy in the last month of life spent $6,925 more on hospitalization expenditure than those who did not ($17,915 vs $10,990). Among decedents seen by a palliative doctor, hospital expenditures in the last month were $8,745 higher compared to those who did not ($16,119 vs $7,374). The study depicted the huge hospitalization spending during the end-of-life for cancer patients in Singapore. Expenditure on end-of-life care can be further reduced with investment in community-based services and integrated care across the community and acute sectors, where hospital-to-home services and earlier palliative care can provide potential cost-effective solutions.

